# Heterotopic pregnancy: A diagnosis we should suspect more often

**DOI:** 10.4103/0974-2700.66563

**Published:** 2010

**Authors:** Karim Ibn Majdoub Hassani, Abderrahim El Bouazzaoui, Mohammed Khatouf, Khalid Mazaz

**Affiliations:** Department of General Surgery, Universitet Hospital Hassan II, Fes, Morocco; 1 Department of Anesthesiology, Universitet Hospital Hassan II, Fes, Morocco

Spontaneous heterotopic pregnancy is a rare clinical and potentially dangerous condition in which intrauterine (IU) and extrauterine pregnancies occur at the same time. It can be a life-threatening condition and can be easily missed, with the diagnosis being overlooked. A high index of suspicion is needed in women with risk factors for an ectopic pregnancy and in low-risk women with an IU gestation who have free fluid with or without an adnexal mass or in those presenting acute abdominal pain and shock. The ectopic component is usually treated surgically and the IU one is expected to continue normally.

**Figure 1:**
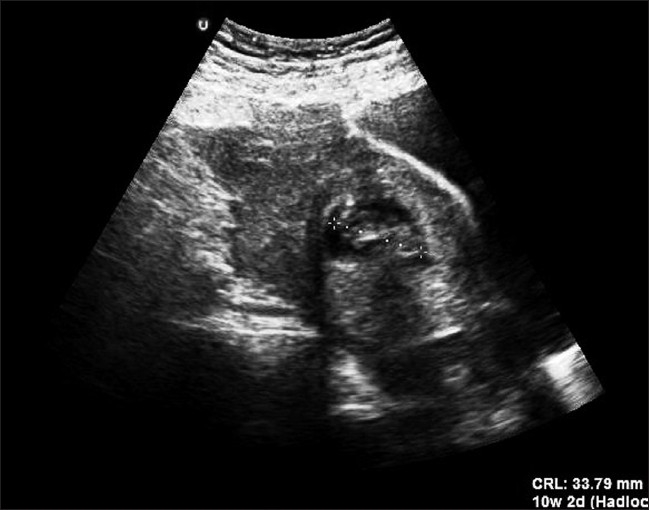
Ultrasonography showing free intraperitoneal fluid and a normal-looking intrauterine gestation with a positive fetal heart rate, consistent with a fetal age of approximately 10 weeks of amenorrhea

**Figure 2:**
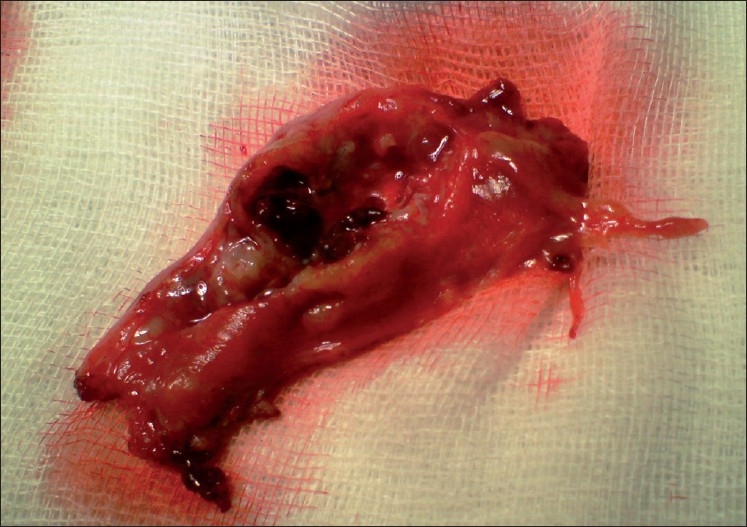
Salpingectomy specimen

A 28-year-old woman, gravid III Para II, was admitted to the emergency department at 10 weeks of amenorrhea, with acute abdominal pain, dyspnea and hypotension. She had no vaginal bleeding. Her current pregnancy occurred spontaneously. This was a spontaneous conception with no previous fertility treatment and she did not use any contraception. Her medical history did not suggest any history of pelvic inflammatory disease, abortions, infertility or abdominal surgery or trauma. The physical examination revealed a conscious woman with discolored conjunctives and cutaneous paleness, systolic blood pressure of 70 mmHg, shortness of breath, profuse sweating and a tachycardia, with a weak and rapid pulse rate of 130 beat per minute. Abdominal examination was suggestive of an acute abdomen with severe tenderness, guarding and rigidity. Laboratory data on admission showed a white blood cell count of 7900 elements/mm^3^, a hematocrit of 18% and serum hemoglobin concentration of 9.1 g/dl, with a normal blood platelet level (390,000/mm^3^), a blood urea of 0.45 g/L and a creatinine level of 10 mg/L. Hemostasis laboratory data, chemistry and serum lipase were within normal limits. The patient was admitted to the intensive care unit (ICU) with a swift assessment of her airway, breathing and circulation. She was provided by taking a central venous line (peripherally inserted central catheter) and initial resuscitation was begun by physiological serum and conventional crystalloid solutions. After hemodynamic stability, an abdominal ultrasonography (US) was realized, which demonstrated free intraperitoneal fluid and a normal-looking IU gestation with a sac of 33.79 mm in diameter and a crown-rump length (CRL) of 28 mm, with a positive fetal heart rate consistent with a fetal age of approximately 10 weeks and 2 days of amenorrhea [Figure 1]. These US findings (available IU pregnancy with a free intraperitoneal fluid) in a hypovolemic-shocked patient with no history of trauma made us think about the presence of a possible concurrent ectopic pregnancy that did not appear in emergency bedside sonography. In addition to this, the patient became acutely hypotensive with an associated increase in abdominal girth. This episode of hypotension was minimally responsive to fluid resuscitation. A stat hemogram confirmed an acute decrease in her hematocrit. It was indispensible to shift the patient to the operating room for an emergency surgery to control the source of bleeding. An emergency exploratory laparotomy was performed under general anesthesia through a subumbilical incision, leading to a finding of a ruptured hetertopic pregnancy. 1.5 L of blood was evacuated from the free peritoneal cavity. There was a 3–4 cm left tubal ectopic pregnancy as well. Both the ovaries appeared normal. A total left salpingectomy was performed [Figure 2] with removal of the hemoperitoneum and peritoneal lavage. The patient was transfused with 8 units of blood during and after the surgery. The post-operative period was uneventful. Histology of the salpingectomy specimen confirmed chorionic villi suggestive of an ectopic pregnancy. An abdominal ultrasound scan was performed on the post-operative day 1, which revealed a viable IU pregnancy. The patient recovered uneventfully and was discharged from the hospital within 4 days. The IU pregnancy proceeded without complications. This pregnancy is currently estimated at 6 months, with satisfactory ultrasound controls, and the patient is free of any symptoms.

**Figure 3:**
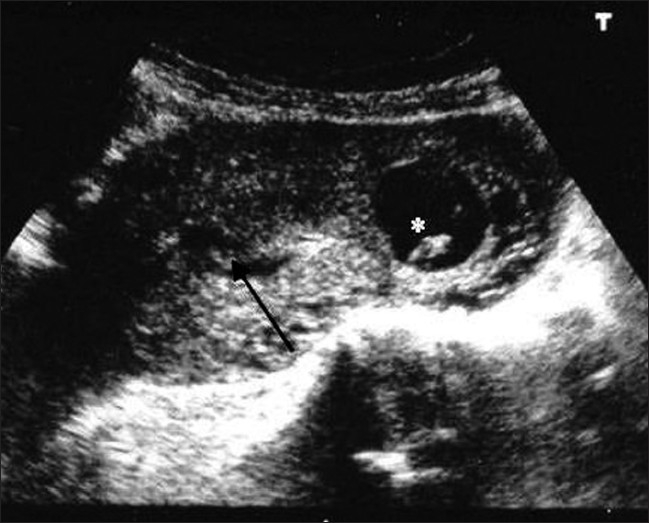
Image of a transvaginal sonography of the uterus (transverse section) showing an intrauterine gestation (black arrow) coexisting with an ectopic cornual pregnancy (*) with a sac of 25 mm in diameter, containing an embryo with a crown rump length of 13 mm

**Figure 4:**
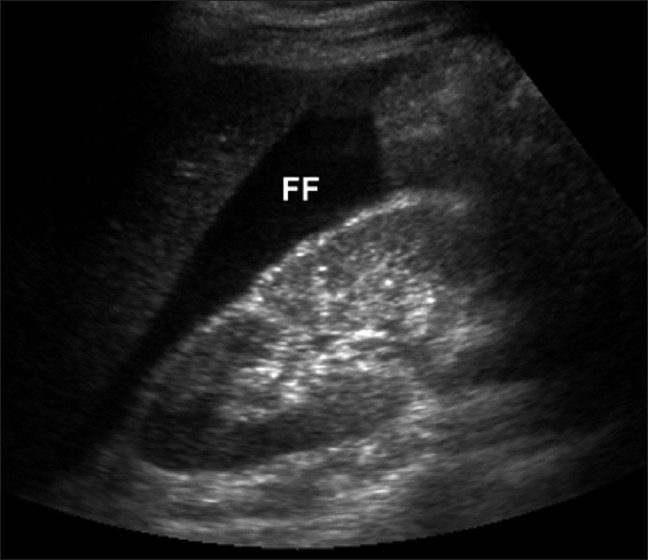
Ultrasonography demonstrating free fluid adjacent to the kidney, consistent with a large amount of hemoperitoneum in a patient with ruptured ectopic pregnancy

Heterotopic pregnancy is defined as the presence of multiple gestations, with one being present in the uterine cavity and the other outside the uterus, commonly in the fallopian tube and uncommonly in the cervix or ovary.[1–3] It was first reported in 1708 as an autopsy finding.[4] In natural conception cycles, heterotopic pregnancy is a rare event, occurring in <1/30,000 pregnancies.[5–7] It occurs in about 0.08% of all pregnancies.[8] With assisted reproduction techniques, however, this incidence increases to between 1/100 and 1/500.[9,10] It occurs in 5% of pregnancies achieved after in vitro fertilization.[11] Spontaneous triplet heterotopic pregnancy has also been reported, with two yolk sacs seen in one tube.[12] In another case, an ectopic pregnancy in each tube with a single IU gestation was reported.[13] Heterotopic pregnancies are usually diagnosed from 5 to 34 weeks of gestation.[14] Tal *et al*.[9] reported that 70% of the heterotopic pregnancies were diagnosed between 5 and 8 weeks of gestation, 20% between 9 and 10 weeks and only 10% after the 11th week. Our case was diagnosed at 11 weeks, when the ectopic pregnancy was ruptured. The early diagnosis of heterotopic pregnancy is often difficult because the clinical symptoms are lacking. Usually, signs of the extrauterine pregnancy predominate.[15] Four common presenting signs and symptoms, abdominal pain, adnexal mass, peritoneal irritation and an enlarged uterus, were defined in the literature.[15] Abdominal pain was reported in 83% and hypovolemic shock with abdominal tenderness, which is the case of our patient, was reported in 13% of heterotopic pregnancies. In addition, half of the patients did not complain of vaginal bleeding in another publication.[9] Vaginal bleeding does occur; however, it may be retrograde from the ectopic pregnancy due to the intact endometrium of the IU pregnancy.[16] The recent advances in transvaginal sonography (TVS) helped in the early diagnosis of heterotropic pregnancy. US, especially transvaginal scanning, has proven to be an invaluable tool in the diagnosis of this condition. However, the sensitivity of TVS in diagnosing heterotropic pregnancy is only 56% at 5–6 weeks.[17] In the TVS of the uterus, the typical image of a heterotopic pregnancy is the presence of an IU gestation coexisting with an ectopic cornual pregnancy containing an embryo [Figure 3].[18] A retrospective study of ultrasonographic images found that a tubal ring (an adnexal mass with a concentric echogenic rim of tissue, a gestational sac, surrounding a hypoechoic empty center) was present in 68% of the ectopic pregnancies in which the tube had not ruptured.[19] If the pregnancy is <6 weeks, diagnosis is the presence of a cardiac activity. At times, even with TVS, the adnexal sac can be mistaken for a hemorrhagic corpus luteum or ovarian cyst, especially in hyperstimulated ovaries.[20] A heterotropic pregnancy goes unnoticed in the presence of IU pregnancy. Therefore, if the beta-hCG (human chorionic gonadotropin) levels are higher for the period of gestation with an IU pregnancy, one must look for a coexistent tubal pregnancy. Sometimes, there are no conclusive adnexal findings and the diagnosis of ectopic pregnancy may be based on other ultrasound features, such as hematoperitoneum, hematosalpinx and free fluid in the peritoneum [Figure 4] or the pelvis; e.g., in the pouch of Douglas.[21] In our case, signs and symptoms of peritonism and shock resulted from internal bleeding secondary to the ruptured ectopic tubal pregnancy. Sometimes, the identification of an IU pregnancy can divert attention from the possibility of a concurrent ectopic pregnancy. However, even if we suspect its existence, its identification in US is usually much more difficult with the presence of a big hemoperitoneum. In the case of an IU pregnancy with acute lower abdominal pain, the possibility of a heterotopic pregnancy should be considered. This condition is very rare in a natural cycle. However, with the increasing use of assisted conception techniques, doctors must be alert to the fact that confirming an IU or ectopic pregnancy clinically or by ultrasound does not exclude a coexisting ectopic or IU pregnancy, respectively. After diagnosis, the ectopic component in case of rupture is always treated surgically and the IU pregnancy is expected to continue normally. In case the ectopic pregnancy was detected early and was unruptured, treatment options include expectant management with aspiration and installation of potassium chloride or prostaglandin into the gestational sac.[22] Systemic methotrexate (MTX) or local injection of MTX cannot be used in a heterotopic pregnancy owing to its toxicity, although some authors have used instillation of a small dose.[23] The laparoscopic approach is technically feasible for both cases without disrupting the course of an IU pregnancy.

In short, doctors must be alert to the fact that confirming an IU pregnancy clinically or by ultrasound does not exclude the coexistence of an ectopic pregnancy that should systematically be suspected in any woman presenting abdominal pain with hypovolemic shock during pregnancy.
